# Protective effects of *Scrophularia striata* in Ovalbumin-induced mice asthma model

**DOI:** 10.1186/2008-2231-21-56

**Published:** 2013-07-09

**Authors:** Abbas Azadmehr, Reza Hajiaghaee, Mohammad Ali Zohal, Ghorban Maliji

**Affiliations:** 1 Immunology department, Qazvin University of Medical Sciences, Qazvin, Iran; 2 Pharmacognosy & Pharmaceutics department of Medicinal Plants Research Center, Institute of Medicinal Plants, ACECR, Karaj, Iran; 3 Internal medicine department, Qazvin University of Medical Sciences, Qazvin, Iran; 4 Immunology department, Mazandaran University of Medical Sciences, Sari, Iran; 5 Immunology department, Babol University of Medical Sciences, Babol, Iran

**Keywords:** Asthma, Allergy, Cytokines, Immunoglubolin E, Scrophularia striata

## Abstract

**Background:**

*Scrophularia striata* Boiss. (Scrophulariaceae) is a plant growing in the northeastern part of Iran and being used as a traditional herb for various inflammatory disorders.

This study was designed to investigate the protective effects of the *Scrophularia striata* extract in Ovalbumin (OVA) induced-asthma mice model.

**Methods:**

OVA-sensitized mice were intrapritonealy treated with two doses (100 and 200 mg/kg) of the extract on days 8 to 14 separately. Broncoalveolar lavage fluids (BALF) was collected 48 h after the final OVA challenge and then the number of eosinophils and other inflammatory cells were assessed by direct microscopic counting. In addition, total immunoglubolin (Ig) E and OVA-specific IgE levels in serum, IL-4 and IL-5 cytokines in BALF were determined by Enzyme-Linked Immunosorbent Assay. Moreover, phytochemical assay by thin layer chromatography (TLC) and the 2, 2 diphenyl-1-picrylhydrazyl (DPPH) were used to evaluate the main compounds and the antioxidant capacity of the plant extract, respectively.

**Results:**

The results showed that the main components; including flavonoids, phenolic compounds and phenyl propanoids were presented in the *S. striata* extract. In addition, the treatment with extract significantly reduced the number of inflammatory cells and suppressed T-helper 2 (Th2) cytokines including IL-4 and IL-5 in BALF. Also, total IgE and OVA-specific IgE levels in the serum decreased.

**Conclusion:**

Collectively, it is concluded that the extract has the potential to modulate the Th2 cytokines and could be used as immunomodulatory agent in the treatment of allergic asthma.

## Introduction

Allergic asthma is a chronic inflammatory disease that recognized by airway inflammation and obstruction, mucus hyper secretion and airway hyper responsiveness [1].

The prevalence of inflammatory and allergic airway diseases such as asthma has significantly increased in recent decades. The asthma is associated to T-helper (Th) type 2 cells response, immunoglobulin (Ig) E-mediated mast cell activation, and other inflammatory factors, including eosinophils, B cells, cytokines and chemokines [2].

In addition, Th2 lymphocytes play important role in initiation and progression of allergic diseases such as asthma through their ability to release interleukin (IL)-4 and IL-5 [3].

On the other hand, IL-4 is important to induce of isotype class switching which is required for B cells to express IgE and also, IL-5 is pivotal for growth, differentiation, recruitment and survival of eosinophils [4,5]. Therefore, most efforts are being made to reduce the inappropriate Th2 response to reduce allergic airway diseases. In this regards, Therapeutic concepts include Th2 cytokine inhibitors; neutralizing antibodies directed IgE, histamine and leukotriene blockers, as well as other targets [6,7].

In recent years, many researches have searched to find novel compounds with greater antioxidant activity. In this regard, natural compounds isolated from medicinal plants can be good candidates to study their antioxidant and anti-inflammatory activities. *Scrophularia striata* Boiss. (Scrophulariaceae) is a plant growing in the northeastern part of Iran being used as a traditional herb for various purposes. Several species of *Scrophularia* have been used since ancient times as sedative in folk medicine and for treatment of illnesses such as scrophulas, scabies, eczema, psoriasis and tumors [8]. Our previous studies in vitro demonstrated the inhibitory effect of *S. striata* extract on nitric oxide and pro-inflammatory cytokines including TNF-α, IL-1β and PGE2 production by macrophages [9,10]. In addition, the anti-inflammatory and immunomodulatory activity of some species of *Scrophularia* has also been shown by other investigators [11-13]. Moreover, several compounds from various *Scrophularia* species with anti-inflammatory and neuroprotective properties including iridoids and phenyl propanoids have been isolated [13]. In another study, flavonoids, phenolic compound, quercetin and isorhamnetin *3-O*-rutinoside with antioxidant activity were also isolated from *S*. *striata*[14]. Given these data, we decided to evaluate the anti-asthmatic effect of *S. striata* extract in Ovalbumin-induced mice asthma model.

## Materials and methods

### Plant material and preparation of the extract

The aerial parts of *S. striata* were collected from Ruin region in northeastern part of Iran, in May 2010 and air dried at room temperature. A sample was authenticated by Dr. Faride Attar, from Tehran University, Faculty of Sciences and a voucher specimen (Herbarium No: 36501) was preserved in the herbarium of the Tehran University Faculty of Sciences, Tehran, Iran. Aerial parts of the plant was dried, powdered (20 g) and macerated with an 80% ethanol solution for 3 days with three changes of the solution. The resulting extract was filtered and evaporated under vacuum into a dried powder extract of *S. striata.* In this study, the extract dissolved in dimethylsulfoxide (DMSO), (CH_3_)_2_SO, (% 0.1 v/v) and then used at appropriate concentrations.

### Phytochemical assay

In order to recognize chemical components of extract, thin layer chromatocheraphy (TLC) was used. A variety of indicators including vanillin sulfuric acid; ferric chloride and natural product polyethylene glycol were used in this assay. The indictors were sprayed on prepared thin layers of extract and were observed at 260 and 280 nm wavelengths under UV light.

### DPPH assay

The DPPH test was used to evaluate the antioxidant capacity of the plant extract [15]. Briefly, one thousand microlitres of various concentrations (250, 125, 62.5, 31.25, 15.62 and 7.81 μg/ml) of the extract of *S. striata* in ethanol was added to 4 ml of 0.004% methanol solution of DPPH. After a 60 min incubation period at room temperature, the absorbance was read against a blank at 517 nm. Inhibition of free radical by DPPH in percent (I %) was calculated in following way:

$$I\%=\left[\left(\mathrm{Ablank}–\mathrm{Asample}\right)/\mathrm{Ablank}\right]\times 100$$

A blank =Absorbance of the control reaction (containing all reagents except the test compound).

A sample =Absorbance of the test compound. Extract concentration providing 50% inhibition (IC_50_%) was calculated from the graph plotted inhibition percentage against extract concentration. IC50% values were compared to IC50% value of a “standard” antioxidant, in this case ascorbic acid (AA), obtained by the same procedure.

### Determination of total phenolic assay

The total phenolic content of dry herbs was determined by using the Folin-Ciocalteau assay [16]. An aliquot (1 ml) of extract or standard solution of gallic acid (20, 40, 60, 80 and 100 mg/L) was added to 25 ml volumetric flask, containing 9 ml of destilled deionised water (dd H2O). A reagent blank using dd H2O was prepared. One milliliter of Folin-Ciocalteu’ sphenol reagent was added to the mixture and shaken. After 5 min, 10 ml of 7% Na2CO3 solution was added to the mixture. The solution was diluted to volume (25 ml) with dd H2O, and mixed. After incubation for 90 min at room temperature, the absorbance against prepared reagent blank was determined at 750 nm. Data of total phenolic contents are expressed as milligrams of gallic acid equivalents (GAE) per gram dry weight (mg _GAE_/g _DW_). All samples were analyzed in duplicates.

### Animals

Six- to 7- weeks old male Balb/c mice were purchased from the Pasteur Institute of Iran (Tehran, Iran). In this study, all the animal experiments were approved and performed according to the guidelines of the Ethical Committee of Institute of Medicinal Plant (IMP). All mice had access to standard laboratory rodent chow and water ad libitum. All procedures involving animals were conducted in accordance with the Guidelines for Laboratory Animal Experiments in IMP Animal Research and Care Center.

### OVA-sensitization challenge and administration of the extract

OVA sensitization and airway challenge were performed as previously described with minor modification [17]. In brief, mice were sensitization on days 1 and 7 by subcutaneously (SC) injection of 100 μg of ovalbumin (Sigma, USA) emulsified in 1mg of aluminum hydroxide (AL (OH) 3) (Merk, USA) as adjuvant in 200 μl of phosphate buffered saline (PBS). The efficiency of sensitization was assessed by measurement of serum total IgE levels and also eosinophilia and total inflammatory cells count on day 8. Then, mice were challenged with intraperitoneal (IP) injection of 10 μg of ovalbumin in 200 μl of PBS on day 14. The mice were divided into five groups, each containing eight mice. The control group (1) received only PBS (Vehicle). The control group (2) treated with AL (OH) 3. Positive control group (3) was immunized by subcutaneous injection of a suspension containing 100μg of ovalbumin and 1mg AL (OH) 3 in 200 μl of PBS on days 1 and 7. The treatment groups (4 and 5) were sensitized by ovalbumin and then intrapritonealy treated with 100 and 200 mg/kg of extract on days 8 to 14 separately.

### Preparation of the bronchoalveolar lavage fluid (BALF) and inflammatory cells count

Lung lavaging was performed 48 h after the last OVA challenge (on day 16) for preparation of bronchoalveolar lavage fluid (BALF). In brief, the thorax cavity of mouse was opened and then sheerd the omohyiod and stylohyoid muscles, then for prevention of lavage reflux, a needle or a fine polyethylene tube was fixed in trachea and 1 ml of PBS was injected to the fixed tube via insulin syrange and then it was aspirated (three time) until 2 ml of BALF was taken. The suspension of BALF was centrifuged and the supernatant collected and stored at −70°C. Inflammatory cell numbers including eosinophil, lymphocyte, neutrophil, macrophage and total cells were determined by direct microscopic counting with a hemocytometer after exclusion of dead cells by trypan blue staining. To determine differential cell counts in BALF, the cells stained using Diff-Quik Stain reagent (B4132-1A; Dade Behring Inc., Deerfield, IL) according to the manufacturer’s instructions.

### Evaluation of inflammation score by lung histology study

For evaluation of inflammation score, 48 hours after OVA-challenge, the lung tissues removed and fixed in 10% neutral buffered formalin at 4°C for 24 h. Tissues were embedded in paraffin, sectioned at 4 μm thickness, and then stained with hematoxylin and eosin (H&E) to estimate inflammation by light microscopy. The degree of cell inflammation in the lung tissue was scored in a double-blind screen by two independent investigators [18].

### Evaluation of total IgE and OVA-specific IgE levels in serum

For evaluation of total IgE and OVA-specific IgE levels, serum samples were collected of the mice on 16 day, respectively. Levels of total mouse IgE and OVA-specific IgE were determined by enzyme-linked immunosorbent assay (ELISA) kits (Serotec, Oxford, UK) according to the manufacturer’s instructions. The absorbance was measured at 450 nm by a micro plate ELISA reader.

### Evaluation of Th2 cytokines including IL-4 and IL-5 in BALF

In order to determine Th2 cytokines, the levels of IL-4, IL-5 in BALF were measured by enzyme-linked immunosorbent assay (ELISA) kits (BioSource International, Camarillo,CA) according to the manufacturer's protocol.

### Statistical analysis

Data represented as mean ± standard deviation. Statistical analyses were performed by one-way analysis of variance (ANOVA) and a post–hoc Bonferroni’s test to express the difference among the groups. All analyses performed using SPSS software16. Data considered statistically significant at *P* < 0.05.

## Results

### Chemical components of extract

Phytochemical assay by thin layer chromatography showed the main components; including phenyl propanoids, phenolic compounds and flavonoids were presented in the *S. striata* extract (Table 1).

**Table 1:** **Phytochemical results of *Scrophularia striata *extract**

**Compounds**	**Reagents**	**Standards**	**Results**
Phenylpropanoids and Terpenoids	Vanillin sulfuric acid	Cinamic acid	+
Phenolic compounds	Ferric chloride	Nepitrin	+
Flavonoides	Natural product reagent	Nepitrin	+

### Antioxidant activity and total phenolics compounds

The results of Free radical scavenging capacities of the extract, measured with DPPH assay, and the ratios (IC50%) AA/ (IC50%) extract are shown in Table 2. They represent the ascorbic acid equivalent of the extract antioxidant capacity, i.e. the amount of ascorbic acid in milligrams equivalent to one gram extract. In addition, Phenolic compounds have been recognized as antioxidant agents, which act as free radical terminators [19] and have been known to show medicinal activity as well as exhibiting physiological functions [20]. The total phenolic compounds of dry herb, respectively as shown in Table 2 with gallic acid as standards.

**Table 2:** **Antioxidant capacity and total phenolics compounds of *S. striata *extract**

**Total Phenolics compounds in dry herb (mg **_**GAE**_**/g **_**dw**_**)**	**DPPH radical scavenging activity, IC**_**50% **_**(mg/l)**	**Ascorbic acid equivalent of the extract antioxidant capacity (mg/g)**
10.96	316.69	29.8

### *S. Striata* extract reduced the infiltration of eosinophils and other inflammatory cells into BALF and lung tissue

**Figure 1:**
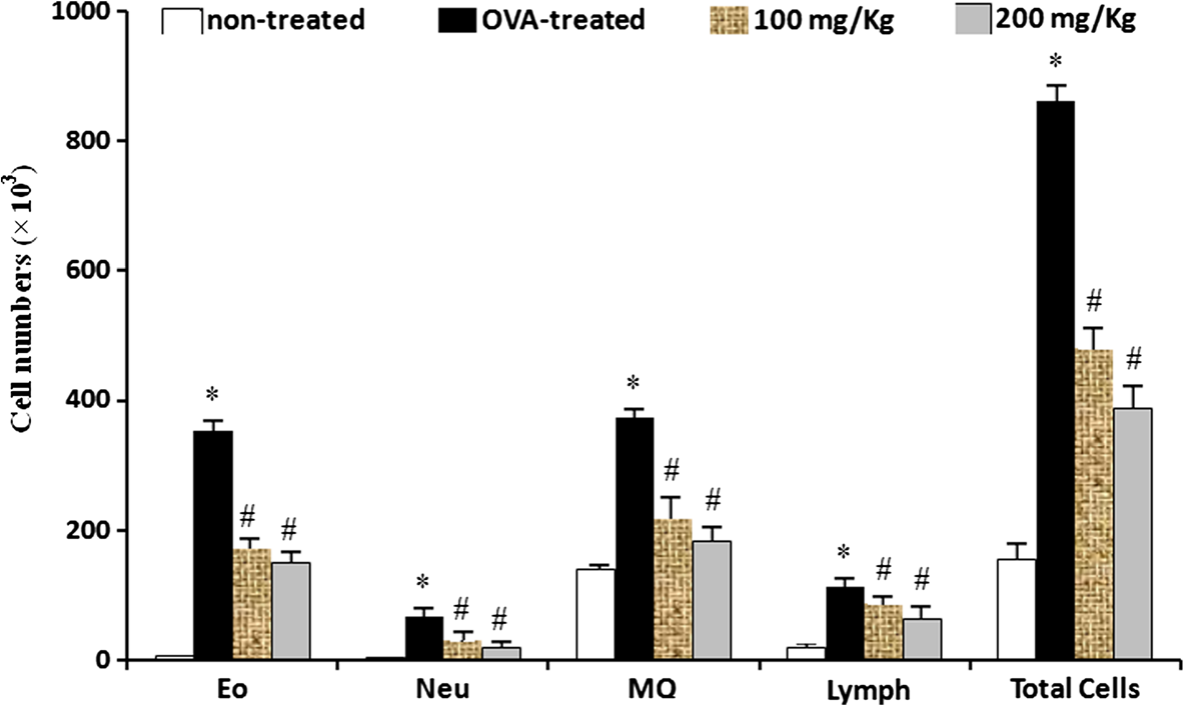
**Effect of *S.striata *extract on the recruitment of inflammatory cells in the Broncoalveolar lavage fluid (BALF) of mice.** The BALF samples were collected 48 h after the last Ovalbumin (OVA) challenge. The numbers of eosinophils and total inflammatory cells were assessed by direct microscopic counting with a hemocytometer. The results showed that the numbers of eosinophils and total inflammatory cells in BALF were significantly decreased in the *S.striata* extract treated mice. * *P* < 0.001 compared with non-treated OVA (Vehicle), # *P* < 0.001 compared with OVA-treated group.

**Figure 2:**
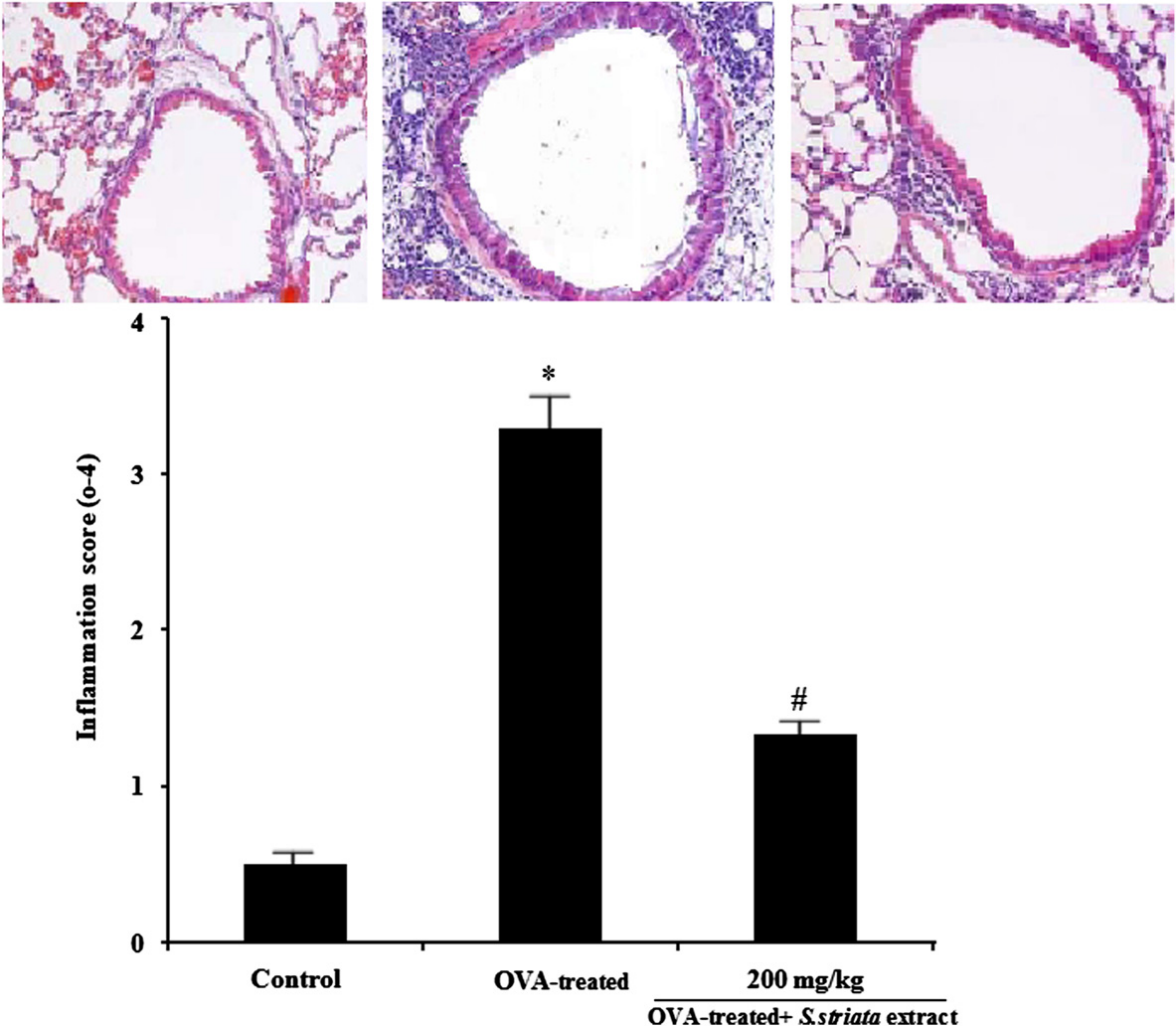
**Effect of *S.striata *extract on the inflammation score in lung tissue.** Lung tissue from animals of respective groups was stained with hematoxylin and eosin and inflammatory cells were studied by light microscopy. Scoring the scope of inflammation by quantitative analysis of inflammatory cell infiltration in lung sections was performed. Inflammation score significantly (P<0.05) reduced in the extract –treated OVA- challenged groups compared with the control OVA- challenged group. * *P* < 0.001 compared with non-treated OVA (Control), # *P* < 0.05 compared with OVA-treated group.

The results of cells number were not shown any significant difference between control groups that received vehicle (1) comparing with control group (2) that received AL (OH) 3. As shown in Figure 1, the numbers of the eosinophils and other inflammatory cells in BALF from the OVA- sensitized or challenged mice were significantly (P<0.05) higher than the negative control mice. However, the infiltration of eosinophils and other inflammatory cells in the BALF significantly decreased in the extract –treated OVA- challenged groups compared with the control OVA- challenged group(P<0.05). Moreover, we determined the suppressive effect of the extract on the infiltration of inflammatory cells in the lung tissue. The results indicated that inflammation score significantly (P<0.05) reduced in the extract –treated OVA- challenged groups compared with the control OVA- challenged group (Figure 2).

### *S. Striata* extract decreased total IgE and OVA-specific IgE in serum

**Figure 3:**
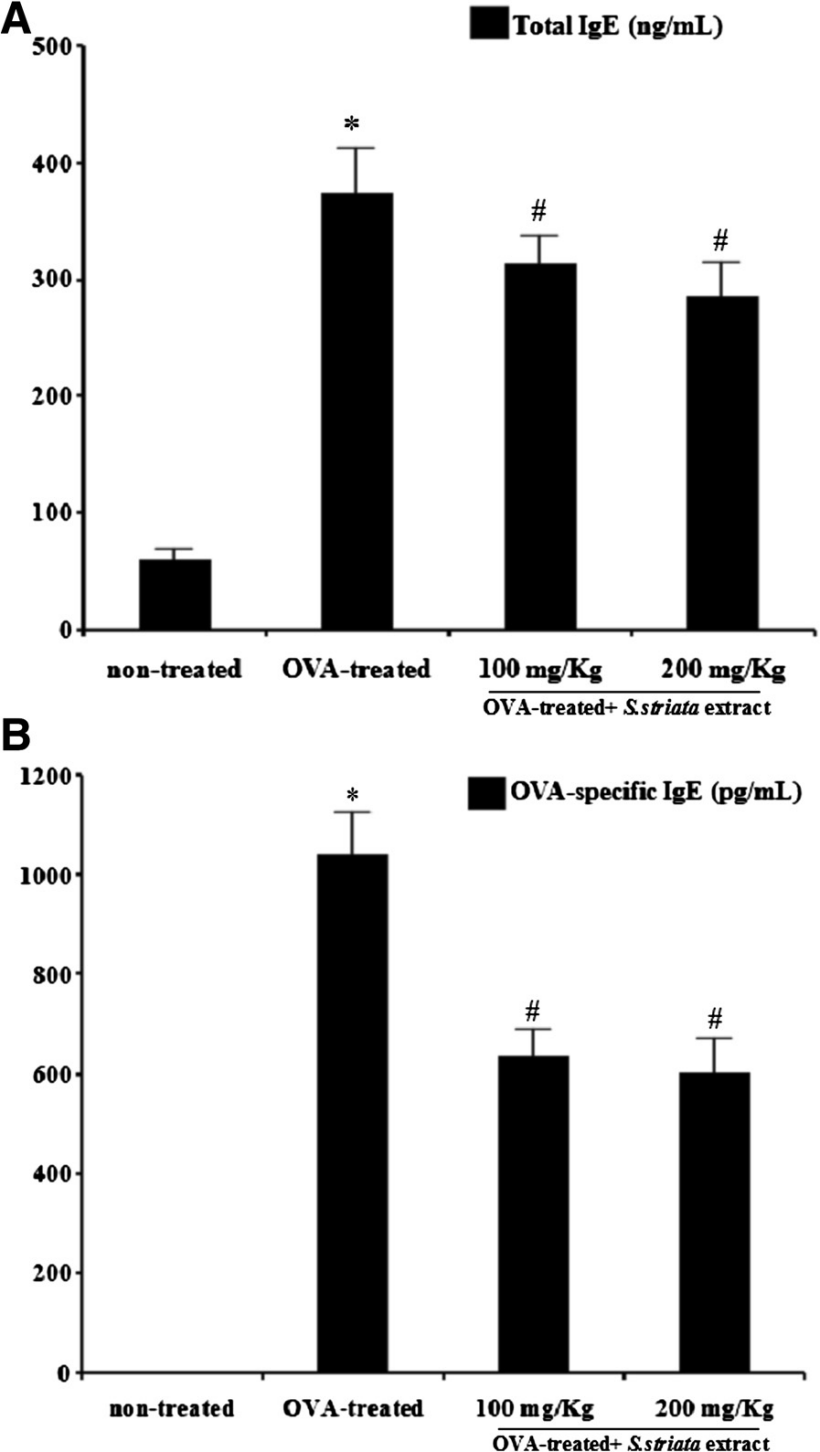
**Effect of *S.striata *extract on the total IgE and OVA-specific IgE levels in serum.** The serum samples were collected 48 h after the last Ovalbumin (OVA) challenge. **(A)** Total IgE level **(B)** OVA-specific IgE level. The results showed that the total IgE and OVA-specific IgE levels in serum were significantly decreased in the *S.striata* extract treated mice. * *P* < 0.001 compared with non-treated OVA (Vehicle), # *P* < 0.001 compared with OVA-treated group.

In order to determine the protective effect of *S. striata* extract on the IgE and antigen specific IgE production, the total IgE and OVA-specific IgE levels in serum were evaluated 48 h after the last OVA challenge. The results were not shown any significant difference between the control groups that received vehicle (1) comparing with control group (2) that received AL (OH) 3. As shown in Figure 3A and B; OVA- sensitized or challenged mice showed significantly (P<0.05) higher levels of IgE (total: 382 ng/ml and specific: 1048 pg/ml) compared with negative control mice (total: 65 ng/ml). In addition, treatment with extract significantly decreased the levels of total IgE and OVA-specific IgE antibodies compared with the control OVA- challenged group (P<0.05).

### *S. striata* extract treatment suppressed the Th2 cytokines in BALF

**Figure 4:**
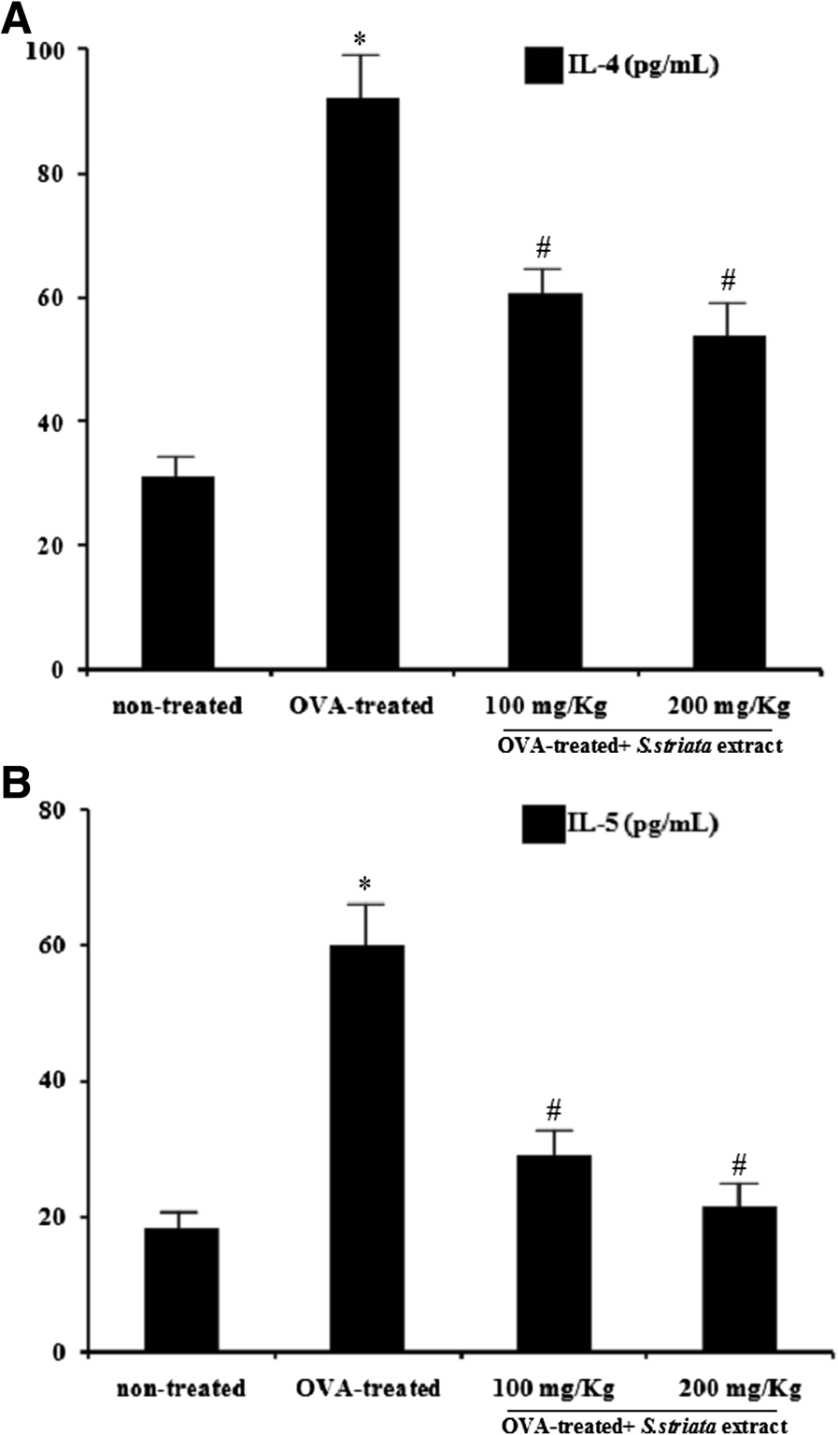
**Effect of *S.striata *extract on the levels of IL-4 and IL-5 in BALF.** In order to determine of Th2 cytokines, the BALF samples were collected 48 h after the last Ovalbumin (OVA) challenge. IL-4 level **(A)** and IL-5 level **(B)**. The results showed that IL-4 and IL-5 levels in BALF were significantly decreased in the *S. striata* extract treated mice. * *P* < 0.001 compared with non-treated OVA (Vehicle), # *P* < 0.001 compared with OVA-treated group.

To evaluate the effect of *S. striata* extract on Th2 cytokines in BALF of OVA- sensitized or challenged mice, IL-4 and IL-5 cytokines were measured by ELISA. The results showed that the levels of IL-4 (94 pg/ml) and IL-5 (60 pg/ml) cytokines in BALF were significantly (P<0.05) increased in OVA- sensitized or challenged mice, while the *S. striata* extract treatment with 200 mg/kg significantly (P<0.05) decreased the levels of IL-4 to 56 pg/ml and IL-5 to 22 pg/ml compared with the control OVA- challenged group as shown in Figure 4A and B.

## Discussion

One of chronic inflammatory disorders is asthma that recognized by airway infiltration of eosinophils and other inflammatory cells, bronchial hyper-responsiveness and airway obstruction [21,22]. On the other hand, in recent decades the prevalence of asthma has significantly increased and extensive efforts have been made to recognize both natural artificial anti-oxidants and anti-asthmatic agents such as medicinal plants. The anti-oxidant, anti-inflammatory and immunomodulatory activity of some species of *Scrophularia* has also been shown by several investigators [9,23-25]. In the present study, we investigated the anti-asthmatic effect of *S. striata extract* in OVA- sensitized /challenged mice asthma model. *S. striata* has been used to treat various inflammatory disorders in animal models. Our previously studies indicated the inhibitory effect of *S. striata* extract on pro-inflammatory mediators production by macrophages including Nitric Oxide (NO), TNF-α, IL-1β and PGE2 and also suppressive effect on matrix metalloproteinases in Wehi-164 tumor cell line in vitro [9,10,25]. The results of this study showed that the treatment with *S. striata* extract (100 and 200 mg/kg) in OVA- sensitized /challenged mice significantly reduced the numbers of eosinophils and total inflammatory cells in the BALF comparing with OVA- sensitized /challenged mice. Th2 cell, a sub-group of lymphocytes, plays an important role in the initiation and progression of allergic asthma by releasing of IL-4 and IL-5 cytokines [26]. Moreover, Th2 response induced airway inflammatory cells infiltration, eosinophil activation, IgE production and mucus secretion [27]. IL-5 cytokine is pivotal for growth, differentiation, recruitment and survival of eosinophils [5]. Our results indicated that *S. striata* extract treatment in OVA- sensitized /challenged mice significantly decreased the levels of IL-4 and IL-5 cytokines in the BALF comparing with OVA- sensitized /challenged mice. The decrease of IL-5 cytokine in the BALF of the mice that had been treated with both extract and OVA may at least in part be responsible for the reduced recruitment of eosinophils. On the other hand, serum IgE level is associated with bronchial asthma and Th2 response [28]. Moreover, IL-4 cytokine is important to the induction of isotype class switching which is required for B cells to express IgE [4]. Our finding in this study showed that treatment with extract significantly decreased total IgE and OVA-specific IgE in the serum of OVA- sensitized /challenged mice that this effect of the extract may at least in part be responsible for the decreased IL-4 cytokine production. In addition, the antioxidant and neuroprotective properties of compounds such as iridoide glycosides and phenylpropanoid esters isolated from *S. buergeriana* have been reported [29-32]. However, in present and previous studies, phenolic compounds, phenyl propanoids and two flavonoids, quercetin and isorhamnetin *3-O*-rutinoside, were identified from *S. striata* extract [14]. Quercetin is well known dietary antioxidants and phenolic compounds are also an anti-inflammatory and antioxidants that the anti-asthmatic and anti-allergic effects of these compounds have been previously reported [33-36]. Whether these compounds are the main responsible compounds for the anti-asthmatic effects of *S. striata* or other compounds are involved in this activity needs further investigation.

In conclusion, the results of this study showed anti-asthmatic and anti-allergic ability of the *S. striata* extract through reducing the IL-4 and IL-5 cytokines production, total and specific-OVA IgE and also decreasing of eosinophils and total inflammatory cells in the BALF of OVA- sensitized /challenged mice asthma model. Further study needs for identifying the main anti-allergic bioactive compounds of this plant and other anti-asthmatic mechanisms.

## Competing interests

The authors declare that they have no competing interests.

## Authors’ contribution

AA: Project design, conducting and supervising experiment, manuscript preparation. RH: conducting experiment and manuscript preparation. MZ, GhM: supervising project. All authors read and approve the final manuscript.
